# Supplementary material for: Genome-Wide Identification and Characterization of Lignin Synthesis Genes in Maize
Source: Int J Mol Sci. 2024 Jun 18;25(12):6710. doi: 10.3390/ijms25126710 (PMC11203529; doi:10.3390/ijms25126710)
Supplement: Supplementary file 1 [file ijms-25-06710-s001.zip › Supplementary FiguresV2.pdf]

# Supplementary Materials

## Genome wide identification and characterization of lignin synthesis genes in maize

**Shuai Wang[1†], Xiaofang Wang[1†], Liangxu Yue[1], Huangai Li[1], Lei Zhu[1], Zhenying Dong[1,2*] and Yan Long [1,2] ***

1 Research Institute of Biology and Agriculture, Zhongzhi International Institute of Agricultural Biosciences, School of Chemistry and Biological Engineering, University of Science and Technology Beijing, Beijing 100083, China.
2 Beijing Engineering Laboratory of Main Crop Bio-Tech Breeding, Beijing International Science and Technology Cooperation Base of Bio-Tech Breeding, Beijing Solidwill Sci-Tech Co. Ltd., Beijing 100192, China.

† These authors equally contribute to this work.

* Correspondence: Zhenying Dong, email: zydong@ustb.edu.cn
Yan Long, email: longyan@ustb.edu.cn

**Citation:** To be added by editorial staff during production.

Academic Editor: Firstname Lastname

Received: date
Revised: date
Accepted: date
Published: date

**Copyright:** © 2024 by the authors. Submitted for possible open access publication under the terms and conditions of the Creative Commons Attribution (CC BY) license (https://creativecommons.org/licenses/by/4.0/).

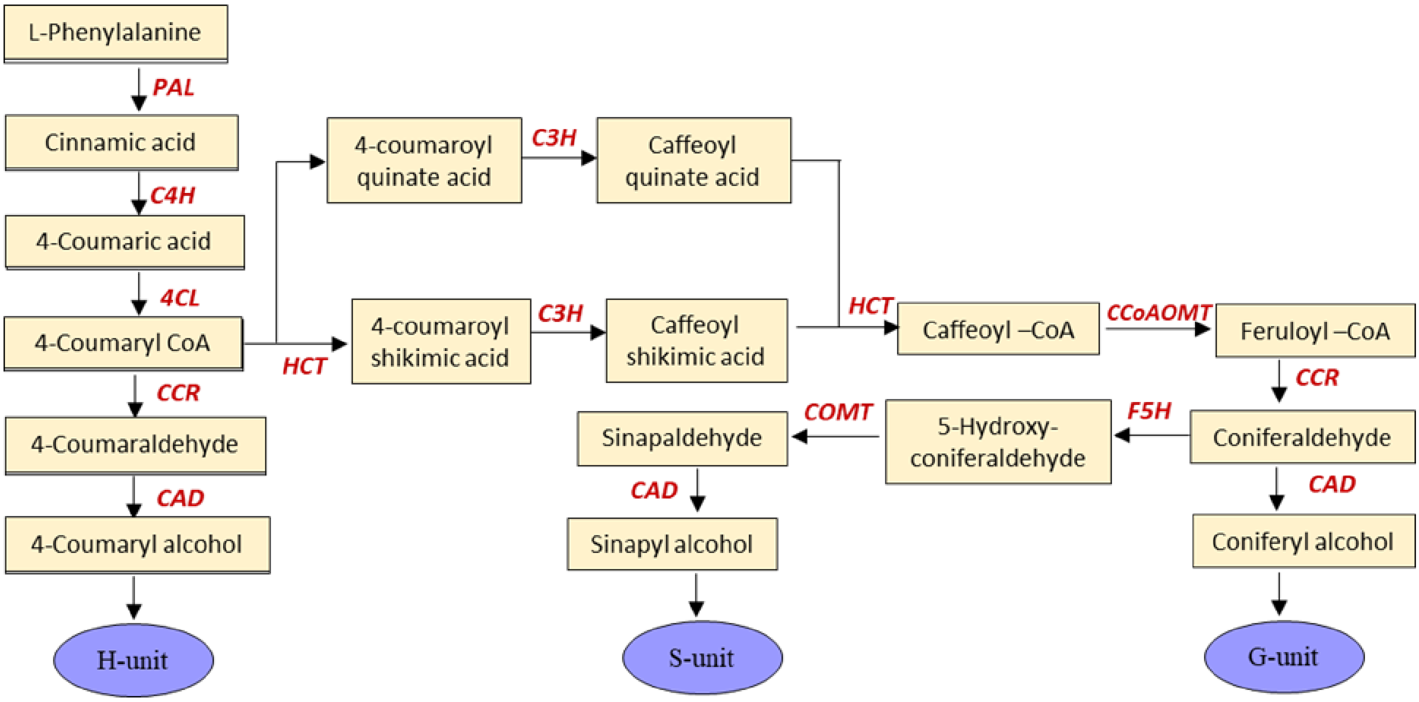


**Supplementary Figure S1.** The biosynthesis pathway of lignin in plants. The characters in the yellow rectangles represent different intermediate products. The characters in the purple ovals mean the three components of lignins. The red characters represent the synthetic genes during lignin synthetic process. PAL: Phenylalanine ammonia-lyase; C4H: Cinnamic acid 4-hydroxylase; 4CL: 4-Hydroxycinnamate CoA ligase; HCT: Shikimate hydroxycinnamoyl transferase; C3H: Coumarate 3-hydroxylase; CCoAOMT: Caffeoyl CoA 3-O-methyltransferase; CCR: Cinnamoyl CoA reductase; F5H: Ferulic acid/coniferaldehyde 5-hydroxylase; COMT: Caffeic acid/5-hydroxyconiferaldehyde 3/5-O-methyltransferase; CAD: Cinnamyl alcohol dehydrogenase.

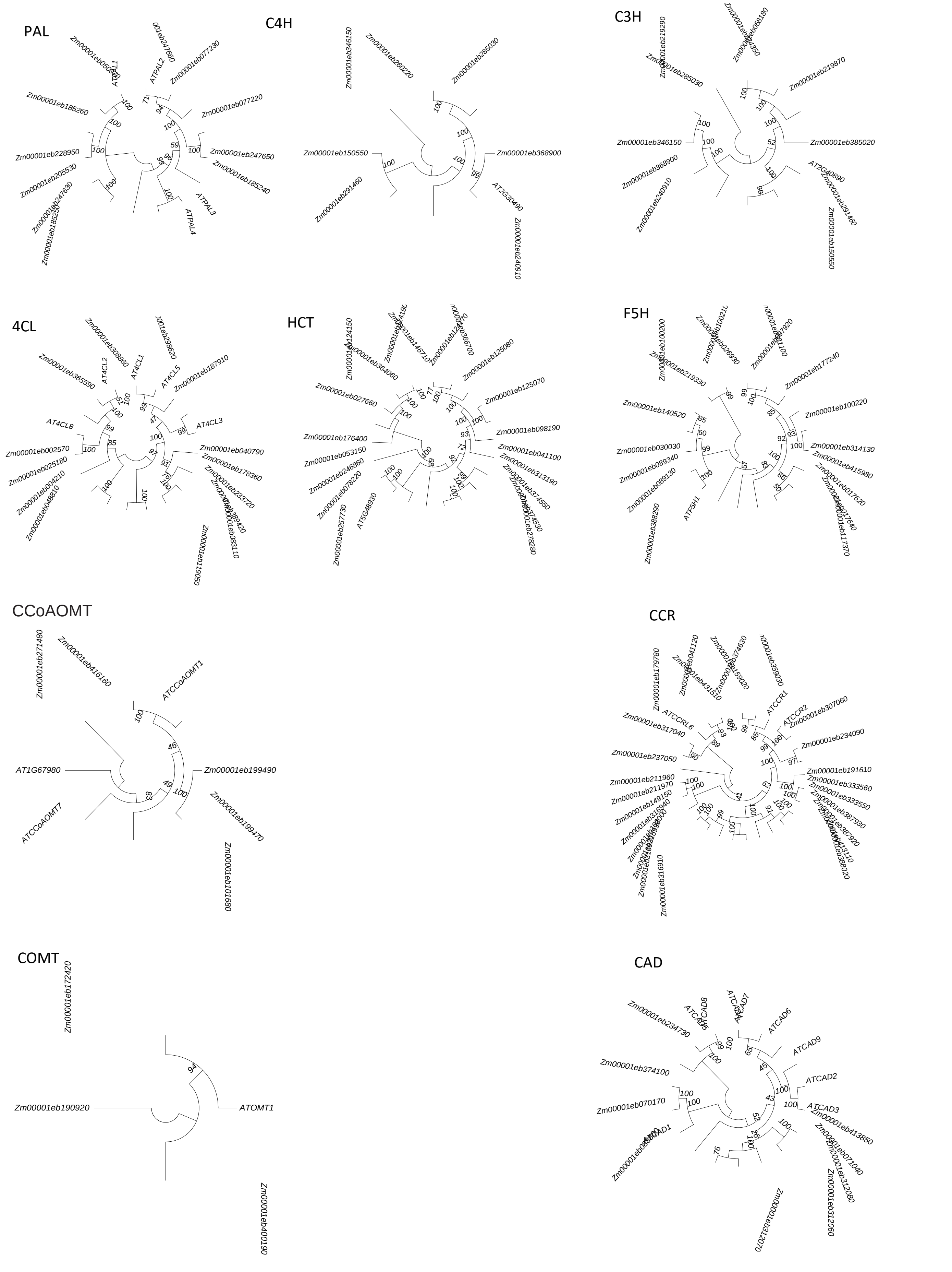


**Supplementary Figure S2.** The phytologic tree between the lignin synthesis genes in the *Arabidopsis* and the maize genome.

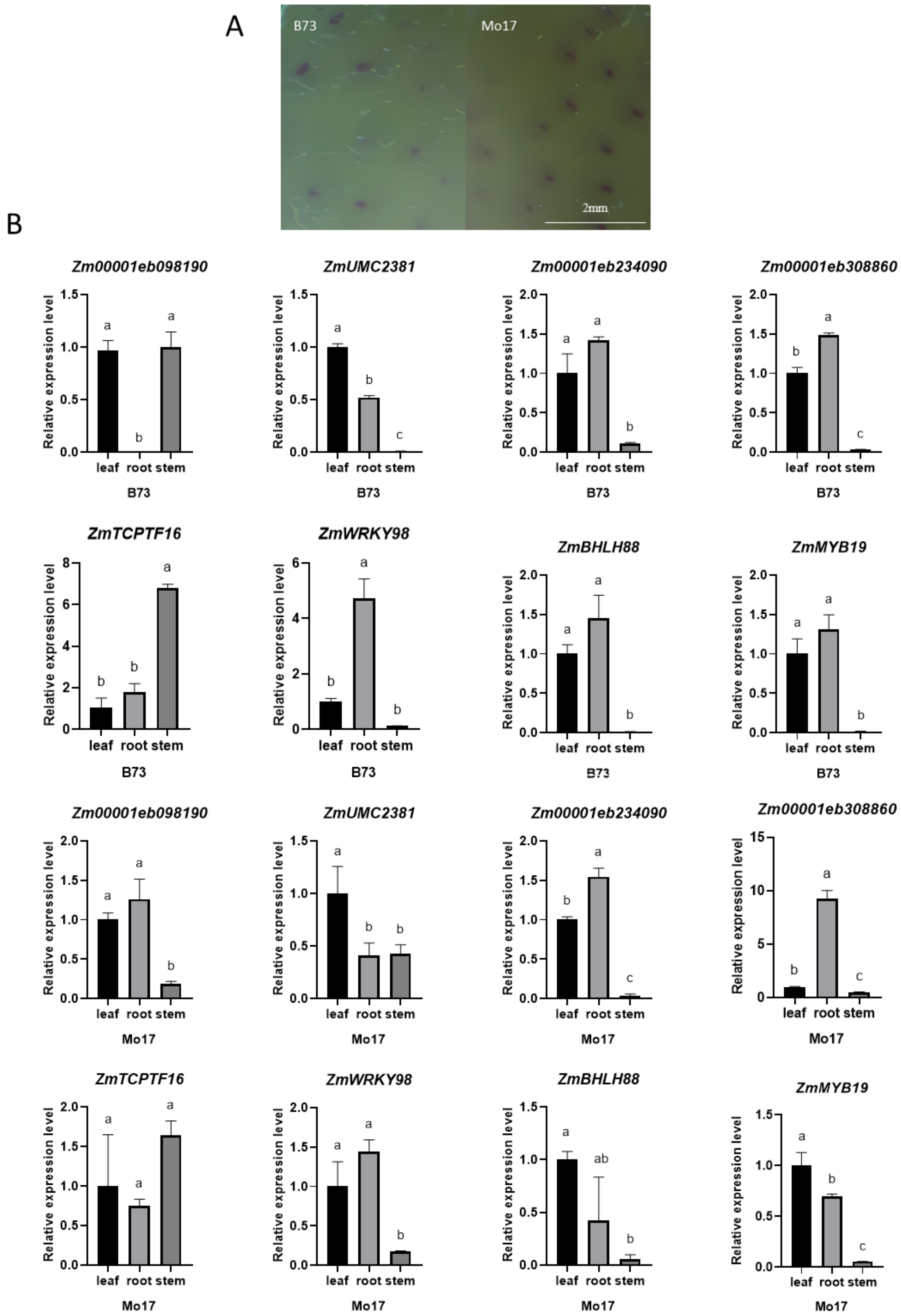


**Supplementary Figure** S3. Expression patterns of candidate genes for stem lignin at 6th-leaf stage. A: Lignin staining of stems of two inbred lines. B: Expression patterns of lignin candidate genes in stems of two inbred line. Data are presented as means ± SE (n = 3). Different letters above line graphs show a significant difference ($p \leq 0.05$) among the two hybrids.
